# Interaction of Plant Extracts with Central Nervous System Receptors

**DOI:** 10.3390/medicines4010012

**Published:** 2017-02-23

**Authors:** Kenneth Lundstrom, Huyen Thanh Pham, Long Doan Dinh

**Affiliations:** 1 PanTherapeutics, 1095 Lutry, Switzerland; 2 Vietnam National Institute of Medicinal Materials, Vietnam Ministry of Health, Hanoi 144, Vietnam; huyenptnimm@gmail.com; 3 School of Medicine and Pharmacy, Vietnam National University, Hanoi 144, Vietnam; longdd.smp@vnu.edu.vn

**Keywords:** medicinal plants, plant extracts and compounds, CNS receptors, in vivo evaluations

## Abstract

**Background:** Plant extracts have been used in traditional medicine for the treatment of various maladies including neurological diseases. Several central nervous system receptors have been demonstrated to interact with plant extracts and components affecting the pharmacology and thereby potentially playing a role in human disease and treatment. For instance, extracts from *Hypericum perforatum* (St. John’s wort) targeted several CNS receptors. Similarly, extracts from *Piper nigrum*, *Stephania cambodica*, and *Styphnolobium japonicum* exerted inhibition of agonist-induced activity of the human neurokinin-1 receptor. **Methods:** Different methods have been established for receptor binding and functional assays based on radioactive and fluorescence-labeled ligands in cell lines and primary cell cultures. Behavioral studies of the effect of plant extracts have been conducted in rodents. Plant extracts have further been subjected to mood and cognition studies in humans. **Results:** Mechanisms of action at molecular and cellular levels have been elucidated for medicinal plants in support of standardization of herbal products and identification of active extract compounds. In several studies, plant extracts demonstrated affinity to a number of CNS receptors in parallel indicating the complexity of this interaction. In vivo studies showed modifications of CNS receptor affinity and behavioral responses in animal models after treatment with medicinal herbs. Certain plant extracts demonstrated neuroprotection and enhanced cognitive performance, respectively, when evaluated in humans. Noteworthy, the penetration of plant extracts and their protective effect on the blood-brain-barrier are discussed. **Conclusion:** The affinity of plant extracts and their isolated compounds for CNS receptors indicates an important role for medicinal plants in the treatment of neurological disorders. Moreover, studies in animal and human models have confirmed a scientific basis for the application of medicinal herbs. However, additional investigations related to plant extracts and their isolated compounds, as well as their application in animal models and the conducting of clinical trials, are required.

## 1. Introduction

Traditional medicine utilizing medicinal plants and plant extracts has been practiced for centuries in parallel to modern Western medicine. Among the more than 300,000 seed plants, some 60% have been utilized for therapeutic interventions [1], particularly in South America [2], Africa [3], and Asia [4]. Thousands of plant extracts have been subjected to the treatment of a wide range of diseases such as obesity [2], rheumatoid arthritis [4], diabetes [5], malaria [6], and not the least for neurological disorders affecting the central nervous system [7,8]. Until recently, the main differences between the therapeutic approaches of the application of traditional medicinal plants and modern drug development has been the former being based on social and ethnic continuity and practical empirical findings, whereas Western medicine has relied on research-based strategies and scientific validation in pre-clinical and clinical settings. Today, a revolution of implementation of scientific approaches and research activities has taken place for medicinal plants. Especially, recent developments in genomics and proteomics research have provided new opportunities for more detailed analysis of plant extracts, improving understanding of the function of their individual components and the effect on human health [9]. Concerning genomics, emphasis on the interaction between medicinal plant genomes and the environment, the correlation between genetic polymorphism, and metabolic diversity have been addressed. Moreover, based on crop plants, it has been possible to expand genomic technologies to medicinal plants for the production of medicinal compounds in plant “factories”. Other approaches include molecular phylogeny and phylogenomics for the prediction of chemo-diversity and bioprospecting.

An important part of the action of medicinal plants targets the central nervous system [10]. Both G protein-coupled receptors (GPCRs) and ion channels possess important functions in the CNS [11,12]. They mediate signal transduction events in the CNS, which are triggered by neurotransmitters and other types of molecules and dysfunction results in inactive, overexpressed or constitutively active molecules, which can cause alterations in ligand binding, receptor desensitization and recycling, potentially resulting in onset of disease [13,14]. There are a large number of studies demonstrating direct effect of plant extracts and/or their compounds on GPCRs and ion channels in the CNS, making them attractive targets for drug development in parallel to conventional drug molecules [15]. In this review, we describe interaction between plant extracts and CNS receptors through several examples.

## 2. Methods of Plant Extract Interactions with CNS Receptors

Studies on the interaction of plant extracts and isolated compounds have been conducted both in vitro and in vivo. In the former case, pharmacological and toxicological evaluations have been carried out in cell lines and primary cells expressing either endogenous or recombinantly expressed CNS receptors [16,17,18]. Generally, radioligand assays have been performed [10] and parallel screening of a number of receptors for the influence of plant extracts on binding properties has taken place [16,17]. Related to in vivo studies, the behavioral effects of interaction of plant extracts with CNS receptors have been evaluated in mouse and rat models [19]. Moreover, plant extracts affecting CNS receptor binding have been subjected to studies on mood and cognition in humans [20]. 

## 3. Effect of Plant Extracts on CNS Receptors and Therapeutic Applications

A number of studies have been conducted on the effect of plant extracts and their isolated compounds on the function of GPCRs and ion channels in the CNS [15]. Several examples are given below on the interaction of plant extracts and CNS receptors (Table 1). Furthermore, the therapeutic and prophylactic potential of plant extracts are presented.

### 3.1. In Vitro Studies

The most common approach has been to analyze the pharmacological properties of CNS receptors in the presence of plant extracts. In this context, water extracts of 10 Chinese herbal drugs were subjected to binding studies on dopamine, muscarinic and serotonin receptors as well as GABA_A_ and benzodiazepine (BDZ) receptors [21]. Not surprisingly, the plant extracts showed binding activity on several receptors (Table 1). For instance, the strongest binding affinity to GABA_A_ receptors was discovered for extracts from *Rhizoma chuanxiong*, *Angelicae sinensis*, and *Gentianae macrophyllae*. In contrast, *Salviae milthiorrhizae* and *Zizphi spinose* showed the highest affinity to BDZ and serotonin 5-HT1A receptors, respectively. Generally, each plant extract interacted with at least two receptors and in several cases with as many as four receptors, indicating the large spectrum of action of plant extracts in the CNS. In another study on Chinese medicinal plants, 18 CNS receptors were evaluated for interaction with 10 plant extracts, which has contributed to the understanding of the action of herbal medicines and has provided bioassays for analysis of active plant ingredients [10]. 

Plant extracts from *Hypericum perforatum*, St. John’s wort, have been frequently studied because of their recognized anti-depressant effects [22]. In one study, *H. perforatum* extracts, fractions, and constituents were subjected to radioligand binding assays on opioid, serotonin, estrogen, histamine, neurokinin, and metabotropic glutamate and GABA_A_ receptors expressed from Semliki Forest virus (SFV) vectors in CHO cells [23]. The lipophilic fraction of *H. perforatum* showed a potent affinity to opioid and serotonin (5-HT6 and 5-HT7) receptors. Furthermore, opioid and serotonin receptor binding was inhibited by *H. perforatum* constituents, including naphthodianthrones, hypericin, pseudohypericin, and hyperforin. The biflavonoid I3,II8-biapegenin inhibited 50% of estrogen receptor binding. GABA_A_ binding inhibition was observed, but did not seem to be specific to St.John’s wort as similar inhibition was observed for extracts from *Valeriana officinalis* and *Passiflora incamata*. The lipophilic fraction of *H. perforatum* showed only low potency of neurokinin-1 (NK1) receptor binding inhibition and no effect on histamine and metabotropic receptors. These findings supported the existence of the proposed synergism of active constituents of St. John’s wort to the anti-depressant effect in the CNS. Similarly, several pure compounds isolated from *H. perforatum* extracts were characterized for interaction with 42 biogenic amine receptors and transporters [16]. Significant binding inhibition was obtained for serotonin 5-HT1D, 5-HT2C, dopamine D3, δ opioid, and BZD receptors by the biflavonoid amentoflavone. Hypericin showed strong activity on dopamine D3 and D4 as well as adrenergic receptors. Only the dopamine D1 receptor interacted with hyperforin. Similarly, leaf and root extracts of the Hawaiian kava, *Piper methysticum*, were evaluated for binding affinity for GABA_A_, dopamine D2, µ and δ opioid, serotonin 5-HT6 and 5-HT7, and histamine H1 and H2 receptors [24]. Binding inhibition was observed for GABA_A_, dopamine, opioid, and histamine receptors, whereas only weak inhibition was detected for serotonin receptors indicating their involvement in pharmacological action of kava extracts. In another approach, whole plant extracts of *Eleusine indica*, *Prunella vulgaris*, *Sophora japonica*, and *Taxillus philippensis* were subjected to fluorescein-labeled ligand binding assays on angiotensin II receptor isolated from mice liver [25]. Relatively potent binding inhibition was observed for extracts from *E. indica* and *P. vulgaris*, whereas the other plant extracts showed only weak or no inhibition. More recently, functional screening of 10 Vietnamese medicinal plants on SFV-based expression of the NK1 receptor in CHO cells demonstrated agonist-induced activity for extracts from *Piper nigrum*, *Stephania cambodica*, and *Styphnolobium japonicum* [26].

Freshly prepared aqueous extracts from *Valeriana officinalis* have been subjected to the effect of binding activity of ionotropic (iGluR) and metabotropic glutamate receptors (mGluR) [27]. Binding studies on rat cortical synaptic membranes demonstrated increase in ^3^H-glutamate binding in presence of valerian extracts. Related to Group II mGluR agents, the *V. officinalis* extract significantly decreased ^3^H-glutamate binding, whereas the opposite was seen for a Group I mGluR agonist. Furthermore, valerian extracts also influenced NMDA and AMPA receptor binding. In a similar study, rat and mouse brain membranes were subjected to binding studies on GABA_A_ and BDZ receptors in the presence of Kava, an intoxicating beverage prepared from *Piper methysticum* (pepper) [18]. However, only weak binding activity was detected for GABA_A_ and BZD receptors, whereas no activity was seen for GABA_B_ receptors. An interesting approach has been to subject homogenates of human cerebral cortical cell membranes to binding studies on nicotinic and muscarinic receptors in presence of *Melissa officinalis*, *Artemisia absinthium* and 3 *Salvia* species [28]. The weak nicotine ligand choline was found in all plant extracts studied. *M. officinalis* extracts showed the highest affinity to nicotinic receptor, while *Salvia elegans* provided the highest affinity to muscarinic receptors. 

According to phytochemical studies, *M. officinalis* contains a number of volatile compounds such as triterpenoids, phenolic acids, and flavonoids [29]. A meta-analysis review on publications on the pharmacological and toxicological effects of crude extracts from *M. officinalis* indicated anxiolytic and antiviral activities as well as influencing mood, cognition, and memory functions. For instance, acetylcholinesterase (AChE) inhibition, stimulation of acetylcholine, and GABA_A_ receptors potentially contribute to the neurological disorders described for plant extracts. In another study, 130 species of medicinal plants identified as Amazonian ethnomedicines for the treatment of cognitive deficiency and dementia were collected in Peru [30]. A total of 228 fractions were screened in 31 radioligand CNS receptor assays and a subset was also screened for functional activity at selected serotonin, muscarinic, and adrenergic receptors. More than 60% inhibition of binding was detected in 91 samples and 135 samples displayed agonist and/or antagonist activity in functional assays. Extracts of *Tanacetum partenium* have been traditionally used as a Danish folk medicine for epilepsy and more recently for prophylactic treatment of migraine [31]. Similarly, methanolic and aqueous extracts of *Valeriana adscendens*, the psychoactive plant traditionally used in the Peruvian Andes for magical-therapeutic rituals, was screened for interaction with CNS receptors [32]. The aqueous extract showed weak affinity for the serotonin 5-HT1A receptor, while both extracts showed affinity for the dopamine D1 receptor. Moreover, no affinity was detected for the serotonin 5-HT2A and 5-HT2C, α1 and α2 noradrenergic, and dopamine D2 receptors.

High affinity binding of GABA_A_ was discovered for a *T. parthenium* ethanol extract, which after fractionation allowed the isolation of apigenin, potentially responsible for the CNS effect. Furthermore, compounds isolated from *Lippa alba* have been subjected to BZD and GABA_A_ receptor binding studies [33]. Luteolin-7-diglucoronide showed the highest affinity with IC_50_ values of 101 µM and 40 µM for BZD and GABA_A_, respectively. In another study, 46 ethanol extracts from 35 species utilized for treatment of mental diseases in South Africa were tested for GABA_A_ receptor binding [34]. High and moderate activity was observed for 7 and 10 extracts, respectively. The strongest GABA_A_ affinity was detected in ethanolic leaf extracts from *Arctopus echinatus*, *Artemisa afra*, four *Helichrysum* species, and *Mentha aquatic*. Wogonin, a flavonoid, has been demonstrated to mediate an anxiolytic effect by allosteric modulation of GABA_A_ receptors [35]. Particularly, flavonoids such as wogonin, baicalein, scutellarein, and baicalin from *Scutellaria baicaliensis* have shown affinity to the BDZ site in GABA_A_ receptors. Furthermore, extracts from the tropical tree *Mitragyna speciosa* (kratom) have been used traditionally in Africa and Southeast Asia for improvement of mood and relief of pain [36]. However, kratom has also been utilized as a recreational drug, which recently has presented some safety concerns following deaths in several countries caused by kratom extracts. The main active alkaloid substances mitragynine and 7-hydroxymitragynine of kratom mediate through monoaminergic and opioid receptors, which require careful monitoring when used for therapeutic purposes.

### 3.2. In Vivo Studies

Plant extracts have been evaluated in a number of in vivo studies, mainly in mice and rat models (Table 1). Oil from SuHeXiang Wan (Storax pili) has been used in Chinese traditional medicine for the treatment of epilepsy [37]. Pre-inhalation of *S. pili* oil was demonstrated to delay the appearance of pentylenetetrazole-induced convulsions, indicating a GABAergic neuromodulation. Furthermore, inhibition of the BZD receptor selective agonist ^3^H Ro15-1788 was observed in rat cerebral cortex. Similarly, oil pre-inhalation significantly decreased GABA levels in mouse brain. The pentobarbital-induced sleeping time was also prolonged. Flavonoids present in medicinal herbs have provided moderate binding affinity at the BDZ site in GABA_A_ receptors mostly in the form of partial agonists in vivo [38]. Structure-activity relationship (SAR)-based approaches have incorporated electronegative groups to the C6 and C3′ on the flavone backbone for significant binding affinity improvement. Furthermore, it has been established that 2′-hydroxyl is a critical moiety on flavonoids triggering the engineering of synthetic flavonoids with improved binding affinity and in vivo activity for therapeutic applications of CNS disorders. An interesting observation revealed that undiluted and saline-diluted potato juice administration (intracisternal or per os) generated anticonvulsant activity in bicuculline-induced seizure threshold test in mice [39]. Potato juice also displaced GABA receptor ligand from binding sites in mice forebrain membranes in vitro. Plant extracts from the Marcgraviaveae family containing betulinic acid have been utilized for treatment of anxiolytic conditions [40]. It was demonstrated in vitro that betulin binds to GABA_A_ receptor sites in mouse brain and competed with the GABA_A_ receptor antagonist bicuculline-induced seizures in vivo.

Experimental models for anti-nociception were evaluated for methanolic extracts of *Neorautenania mitis* in mice and rats [19]. Mice were subjected to acetic acid-induced abdominal constriction, analgesy-meter and Randall-Selitto test, hot-plate test and formalin-induced pain in rats. Extract doses of 5, 10, and 20 mg/kg body weight generated dose-dependent anti-nociceptive activity. The central anti-nociception is suggested to be mediated by opioid receptors. In another approach, the NMDA receptor antagonist memantine and the anti-oxidant and anti-inflammatory agent tea polyphenol were evaluated individually or in combination for neuroprotection in a mouse excitotoxic injury model [41]. Locomotor activity was assessed and brain synaptosomes were harvested. Treatment with memantine significantly rescued mitochondrial function and tea polyphenol decreased the enhanced production of synaptosomal reactive oxygen species (ROS). However, only the combined treatment was able to protect mice against excitotoxic injury providing potential therapeutic applications for brain trauma, brain ischemia, epilepsy and Alzheimer’s disease. Extracts from *Rollinia mucosa* leaves were analyzed for BDZ receptor binding by in vitro autoradiography and single administration in mice [42]. The study revealed reduced binding activity in the hippocampus (29%), amygdala (26%) and temporal cortex (36%).

Although marine plants possess various pharmacological features, they have not been described to provide neuropharmacological efficacy. The polyphenol-rich brown seaweed *Ecklonia cava* was subjected to anti-convulsive and sleep-inducing effects in mice with picrotoxin-induced seizures [43]. Utilizing the phlorotannin-rich fraction (PTRF) from *E. cava* generated depressive effects in the CNS by positive allosteric modulation of phlorotannins on GABA_A_-BZD receptors similar to diazepam (DZP). *E. cava* therefore presents an attractive target for treatment of neuropsychiatric disorders including anxiety and insomnia.

The effect of phytocannabinoids was studied in an experimental autoimmune encephalitis (EAE) model of multiple sclerosis in mice [44]. Sativex®, a combination of ∆(9)-tetrahydrocannabinol botanical drug substance (∆(9)-THC-BDS) and the licensed cannabidiol-botanical drug substance (CBD-BDS) was administered intraperitoneally as well as the individual components separately. The combination therapy significantly improved the neurological deficits in EAE mice and reduced the number and extent of cell aggregates in the spinal cord. Similar results were achieved for treatment with ∆(9)-THC-BDS, but not for CBD-BDS. Furthermore, it was demonstrated that a complete reversion of neurological benefits were detected after selective blocking of the cannabinoid 1 receptor (CB1) with a selective CB1 receptor antagonist. In another study, the South East Asian traditional medicine from the sacred lotus flower extracts (*Nelumbo nucifera*) was subjected to in vitro cannabinoid and opioid receptor radioligand binding assays, functional GTPγS assays and in vivo behavioral evaluations in mice [45]. The *N. nucifera* compounds *O*-methylcoclaurine, *N*-methylcoclauirine, coclaurine, and neferine displayed various degrees of opioid receptor affinities in vitro. In vivo, *N*-nor-nuciferine and asimilobine displayed actions measured by locomotion, catalepsy, body temperature and nociception. Flowers of the Dopati (*Impatiens balsamina*) garden plant have been used as folk medicine for pain and burns in Bangladesh [46]. Methanol extracts of *I. balsamina* have been evaluated for anti-nociceptive activity in chemical- and heat-induced pain models. Plant extracts generated strong dose-dependent anti-nociceptive activity verified by acetic acid-induced writhing, hot plate, tail immersion, and formalin tests. Application of the opioid antagonist naloxone confirmed the association of the Dopati extract with opioid receptors. These findings will encourage further investigations on applying these plant extracts for the therapeutic intervention of different pain-related conditions.

An important issue in relation to CNS drugs is their capacity to penetrate the blood-brain barrier (BBB), which has been extensively studied for plant extracts and their isolated compounds. In this context, the BBB pharmacokinetic permeability properties for the plant *N*-alkylamide pellitorine were evaluated in rats [47]. Intravenous injection of pellitorine showed rapid and high BBB permeation resulting in 97% of pellitorine reaching the brain and only 3% remaining in brain capillaries. In another study, the *N*-alkylamide spilanthol was investigated for BBB permeability in mice showing high and rapid permeation from the blood into the brain [48]. Furthermore, brain penetration of five active isoflavones, puerarin (PU), 3′-methoxypuerarin (MPU), 3′-hydroxypuerarin (HPU), daidzein (DA), and daidzein-8-C-apiosyl-(1-6)-glycoside (DAC), from the roots of *Pureraria lobata* was evaluated in rats using an ultra-fast liquid chromatography tandem mass spectrometry method [49]. All isoflavones except HPU showed rapid BBB penetration, which might explain the neuro-pharmacological properties associated with *P. lobata*. Another aspect concerning plant extracts and their influence of BBB relates to the ability of *Ephedra sinica* extract to block the activity of pathways of complement C3 [50]. It was demonstrated that *E. sinica* extracts alleviated BBB disruption and brain edema in Sprague-Dawley rats with early brain injury. The improved neurological functions were associated with the inhibition of complement C3. In another study, pretreatment with α-tocopherol and ascorbic acid showed strong protection of the BBB exposed to cigarette smoke extract [51]. Furthermore, it was demonstrated that resveratrol, lipoic acid melatonin and coenzyme Q10 inhibited the BBB endothelial release of pro-inflammatory IL-6 and IL-8 cytokines. An interesting observation relates to the BBB permeability of extracts and their compounds from St. John’s wort [52]. For instance, acylphloroglucinol hyperforin, amentoflavone, naphthodianthrones hypericin, and pseudohypericin pass the blood-brain barrier poorly in animals, which might contribute to the inferior effect of these plant compounds on neurotransmitter receptors. 

### 3.3. Clinical Trials and Therapeutic Applications

Although a number of plant extract-based drugs fall into the category of traditional medicines, which have not been subjected to strict clinical trials, the influence of modern Western medicine has encouraged more rigid investigations on the safety and efficacy in human subjects. In this context, the herbal medicine based *Melissa officinalis* extracts traditionally used for memory enhancement, but more widely as a mild sedative and for treatment of insomnia, was evaluated in healthy volunteers [20]. The initial screening included studies on AChE inhibition and cholinergic receptor binding. The cognitive mood was assessed in 20 young healthy individuals receiving doses of 600, 1000, and 1600 mg of encapsulated dried leaves of *M. officinalis*. In vitro studies on human cerebral cortex tissue revealed interaction of the extract with nicotinic and muscarinic receptors. In human volunteers, notable cognitive and mood effects in the form of improved memory performance and increased calmness were observed at all tested time points for the highest concentration, which may prove valuable for combination therapy of Alzheimer’s disease. Importantly, variations in properties were discovered in different preparations from the same plant depending on which extract preparation method was applied. The roots of the “Indian Ginseng” (*Withania somnifera*) have demonstrated some neuroprotective efficacy [53]. For instance, standardized extracts of *W. somnifera* have been shown to possess multidimensional neuromodulatory properties both in vitro and in animal models in vivo. Based on these studies, *W. somnifera* extracts are suggested to target neurotrophic factors, cytoskeletal elements, cell adhesion molecules, and synaptic proteins. To further explore *W. somnifera* extracts in such neurodegenerative diseases as Alzheimer’s disease and Parkinson’s disease, clinical trials need to be conducted. A meta-analysis study comprised eight clinical trials applying traditional *Salvia officinalis* and *Salvia laandulaefolia* remedies for memory enhancement and improved cognitive function [54]. Healthy volunteers were evaluated in six studies for cognitive performance, and in two trials, patients with Alzheimer’s disease were enrolled. The plant extracts exerted beneficial effects through enhanced cognitive performance in both healthy individuals and patients with dementia or cognitive deficiency. The clinical trials demonstrated high safety standards. However, differences in utilization of raw materials and preparations of plant extracts and oils might influence the results and the methodological standards for clinical trials need to be addressed.

Medicinal plants have been used for the treatment of multiple sclerosis. In this context, the potential therapeutic efficacy of hemp seed, evening primrose oil, and Hot-nature dietary intervention has been investigated in relapsing-remitting multiple sclerosis [55]. In a randomized double blind clinical trial, 100 multiple sclerosis patients received hemp seed and evening primrose oils with advised Hot-nature diet, which resulted in significant improvement in relapse rates confirmed by immunological findings. Another approach for treatment of multiple sclerosis has involved cannabinoids with an increasing amount of evidence emerging from anecdotal reports of symptomatic improvement [56]. However, treatment of other symptoms such as tremor and nystagmus has failed to provide any benefit of cannabinoids. The safety profiles of cannabinoid treatment are good with no serious safety concerns, which have encouraged focus on methodological issues and treatment delivery. Furthermore, experimental findings have indicated that cannabinoid-based therapy can promote anti-inflammation, remyelination, and neuroprotection. In another safety trial, the bioavailability of the food-grade formulation of natural curcumin with high bioactivity and BBB permeability was subjected to a randomized double-blinded and placebo-controlled study on 60 subjects suffering from occupational stress-related anxiety and fatigue [57]. The outcome was high safety, good tolerance, and enhanced efficacy in comparison to unformulated standard curcumin. Moreover, a significant improvement in the quality of life with a considerable reduction in stress, anxiety, and fatigue was observed.

The commercially available extracts of St. John’s wort, Texx 300, and Jarsin 300 were compared to placebo in a single blinded study in healthy young volunteers [58]. Both extracts showed a decrease in the cognitive potential P300, suggesting enhanced mental performance, and the observed neurophysiological changes were in line with the proposed clinical efficacy. Moreover, quantitative EEG allowed discrimination between St. John’s wort extracts with respect to time of effect and profile changes on neuronal communication structure. In another study, a dry extract of *Ginkgo biloba* leaves were evaluated on elderly subjects diagnosed with Alzheimer’s disease [59]. The open, uncontrolled trial in 18 patients with light to moderate dementia induced pharmacological effects as established by QPEEG measurements in the CNS similar to previous findings in healthy young volunteers. Typical cognitive activator CEEG profiles were observed after administration of 240 mg of *G. biloba* extract in 8 of 18 patients. Dried rhizome and roots of *Carapichea ipecacuanha* prepared as syrup of ipecac was compared to apomorphine as an emetic in poisoning cases in a prospective, randomized study [60]. Emesis was induced in 87% of ipecac-treated patients and in 77% of apomorphine-treated individuals. One patient suffered from moderate CNS depression in the ipetec-group, whereas significant depression developed in eight patients treated with apomorphine.

Several drugs based on medicinal plants have been approved for clinical use. For instance, Sativex^®^, the cannabinoid-based medicine, has been evaluated for symptomatic improvement in patients with moderate to severe multiple sclerosis related spasticity [61]. Clinical trials have indicated that long-term clinical benefit can be maintained despite disease progression. No safety or tolerability issues have emerged, and no evidence of driving impairment, incidence of falls, or other adverse events have been documented after more than two years of Sativex^®^ use. As indicated above, the St. John’s wort-based drugs Texx 300 and Jarsin 300 have been approved already some time ago [59]. Similarly, *G. biloba* extracts have been approved in Germany and in the US and nowadays used widely globally [60]. 

## 4. Discussion

Medicinal plants and their extracts have traditionally been applied for the treatment of various diseases not the least related to neurological disorders involving CNS receptors. The long tradition of applying medicinal herbs has been mainly based on social and ethnic continuity and treatment efficacy has mainly relied on anecdotal reports and empiric documentation. In contrast, modern Western medicine has relied strongly on research-scientific based approaches including thorough clinical evaluations and safety assessments of candidate drugs before being approved on the market. More recently, there has been a stronger demand for also subjecting medicinal herbs to clinical trials. Indeed, a number of clinical trials have been conducted [20,53,54,55,56,57,58,59,60] for medicinal herbs and plant extracts, which surely has and will in the future further increase the credibility of plant-based drugs. However, among the 39 new drugs approved by the US Food and Drug Administration (FDA), only two were described as botanical drugs [62]. In this context, the first FDA approved plant-based drug, Veregen, a green tea leaf extract used as a topical cream for perianal and genital condyloma, has been critically evaluated for the establishment of a Checklist for New Drug Application of Herbal Medicines for future development of clinical trials on herbal medicines [55]. In Japan, the traditional herbal Kampo medicines, based on Chinese ancient medicines, are prescribed in general practice by physicians and sold as over-the-counter (OTC) formulations for self-medication [63]. Today, 294 Kampo formulations are listed in Japan.

Another issue of importance has been the pharmacological characterization of plant extracts and the identification of fractions and compounds responsible for the interaction with CNS receptors (Table 1). Efficient expression of recombinant receptors and the development of particularly fluorescence-based methods for receptor binding and functionality have facilitated the identification of the interaction of a large number of CNS receptors with various plant extracts. It seems that this interaction is quite complicated with individual plant extracts targeting several GPCRs in parallel, particularly those derived from native tissues. In this context, additional characterization of receptor-plant extract interaction, especially deploying recombinant receptors will enhance the application range of medicinal plants. 

Another difficulty in developing plant-based medicines relates to the variation in concentration of compound contents isolated from plants. For instance, it was demonstrated that samples from *Ligularia fischeri* leaves collected in summer (June) showed higher phenolic compounds, stronger anti-oxidative, and anti-microbial activity than samples collected in winter (December) [64]. Moreover, the location plays an important role as demonstrated for the differences in potency of NK1 receptor inhibition for Vietnamese plants collected from different parts of the country [26]. Furthermore, fruit from high-bush blueberries (*Vaccinium corymbosum*) and low-bush blueberries (*Vaccinium angustifolium*) showed differences in the contents of anthocyanins and total phenolic compounds [65]. It was also demonstrated that the method of extraction influenced the composition of fruit extracts with the highest anthocyanins and total phenolic contents and anti-oxidant capacity observed in extracts using a solvent of acidified aqueous methanol. Additionally, low-bush blueberries showed consistently higher concentrations of anthocyanins, total phenolics, and anti-oxidant capacity in comparison to high-bush blueberries. For these reasons, special attention needs to be paid to addressing the drug manufacturing process of plant raw materials and extracts, which obviously are less straightforward than used for chemically synthesized drug molecules.

## 5. Conclusions 

In summary, the popularity of medicinal plants seems to continue in modern time. Science-based evaluation of plant extracts and characterization of their function has significantly improved our understanding of their use in medicine. In this context, plant extracts and their isolated compounds have demonstrated significant influence on a variety of CNS receptors including activation and inhibition based on in vitro and in vivo preclinical experiments and also in clinical trials (Table 1). The effect of medicinal plants on CNS receptors triggers various brain functions, which provides attractive opportunities for treating neurological disorders and other diseases related to the CNS. However, these studies have also revealed the complexity of the receptor-plant extract interaction where plant extracts can simultaneously inhibit the function of a number of non-related receptors. In this context, application of genomics and proteomics approaches will further improve the understanding. Very importantly, sincere efforts need to be dedicated to thorough pre-clinical studies on medicinal herbs and particularly well-designed and well-executed clinical trials, which in the case of favorable outcome will significantly enhance the credibility of developing plant-based drugs. 

Today, a huge number of plant-based drugs and remedies are used in traditional medicine, but quite a few have also been approved by the health authorities around the world. To further support the application of medicinal plants in treatment of various diseases, additional basic research on receptor-plant extract interaction is needed. Significant efforts are also required for safety and efficacy demonstration of medicinal plants in controlled clinical settings. Most importantly, there needs to be a genuine communication between representatives of traditional plant-based medicines and scientists involved in modern research-based drug development. Only then can the better of the two worlds be accessed, providing the means of developing novel and more efficient drugs. 

## Figures and Tables

**Table 1:** Examples of interaction of plant extracts with CNS receptors.

Receptor	Plant/Extract	Host	Effect/Response	Ref.
5-HT1A	*R. cimicifugae*, *Z.spinose* *V. adsendens*/methanolic, aqueous	Cell lines Cell lines	Receptor affinity Weak affinity, only aqueous extract	[21] [32]
5-HT1D	*H. perforatum/*amentoflavone	Cell lines	Receptor binding	[16]
5-HT2	*H. perforatum/*amentoflavone	Cell lines	Receptor binding	[16]
5-HT6/7	*H. perforatum/*lipophilic fraction *H. perforatum/*hypericin, hyperforin	Cell lines Cell lines	Receptor binding Receptor binding	[23] [23]
AR	*H. perforatum*/amentoflavone, hypericin	Cell lines	Receptor binding	[23]
AT-II	*E. indica, P. vulgaris*	Cell lines	Receptor binding	[25]
BZD	*S. milthiorrhizae*, *Scutellariae* *H. perforatum/*amentoflavone *P. methysticum/*Kava *R. mucosa* leaves *E. cava/*phlorotannin	Cell lines Cell lines Rodent brain Mouse brain Mouse brain	Receptor affinity Receptor binding Weak receptor binding Reduced binding Receptor binding	[21] [16] [18] [42] [43]
CB1	Sativex®	EAE mice	Therapeutic potential	[44]
Estrogen	*H. perforatum/*I3 II8-biapegenin	Cell lines	Receptor binding	[26]
NK1	*P. nigrum*, *S. cambodica*, *S. japonicum* *H. perforatum*/lipophilic fraction	Cell lines Cell lines	Inhibition of NK1 Weak binding	[24] [23]
GABA_A_	Chuanxiong/*R. chuanxion* Danggui/*A. Sinensis* Qiunjiao/*G. macrophyllae* Marcgraviaceae/betulin *S. tuberosum*/juice *L. alba*/polyphenols, iridoids Flavonoids *A. echinatus, A. afra, M. aquatica* *S. baicalensis*/wogonin *P. methysticum/*Kava *E. cava/*phlorotannin *P.methysticum*	Cell lines Cell lines Cell lines Mice, in vivo Mice, in vivo Cell system Mice, in vivo Cell lines Mice, in vivo Rodent brain Mouse brain Cells	Receptor affinity Receptor affinity Receptor affinity Anticonvulsant action Anticonvulsant action Antioxidant, neurosedative Receptor affinity GABA_A_ binding GABA_A_ binding Weak receptor binding Receptor binding Receptor binding	[21] [21] [21] [40] [39] [33] [38] [34] [35] [18] [43] [24]
mAChR	*S. pili* oil	Rat	Delay of convulsion	[37]
mGluR	*T. parthenium*/apigenin		GABA_A_ binding	[31]
NMDA	*S. pill* oil *V. officinalis* Memantine, tea polyphenol *M. officinalis* *M. officinalis*, *A. absinthium*	Cell lines Cell lines Mice, in vivo Humans Human brain	Inhibition of convulsion Receptor binding interaction Neuroprotection Cognition, mood Dose-dependent affinity	[10] [27] [41] [20] [28]
D1	*H. perforatum/*hypericin	Cell lines	Receptor binding	[23]
D3	*H. perforatum*/amentoflavone	Cell lines	Receptor binding	[23]
Opioid	*H. perforatum*/hypericin *H. perforatum*/amentoflavone,hypericin *P. methysticum* *H. perforatum/*amentoflavone *H. perforatum/*lipohilic fraction *H. perforatum/*hypericin, hyperforin *M. speciosa/*mitragynine, 7-HM *N. mitis/*methanolic extracts *N. nucifera* *I. balsamina*	Cell lines Cell lines Cell lines Cell lines Cell lines Cell lines Humans Mice in vivo Mice in vivo Mice in vivo	Receptor binding Receptor binding Receptor binding Receptor binding Receptor binding Receptor binding Mood, pain, drug abuse Anti-nociception in vivo Radioligand binding Anti-nociception in vivo	[23] [16] [25] [16] [23] [23] [36] [19] [45] [46]

5-HT, serotonin 5-hydroxytryptamine receptor; 7-HM, 7-hydroxymitrogynine; AR, adrenergic receptor; AT-II, angiotensin-II receptor; BZD, benzodiazepine; D1, dopamine 1 receptor; GABA_A_, γ-aminobutyric acid receptor; mAChR, muscarinic acetylcholine receptor; NK1, neurokinin-1 receptor; NMDA, *N*-methyl d-aspartate receptor.
